# Cardio-Metabolic Disorders in Non-Alcoholic Fatty Liver Disease

**DOI:** 10.3390/ijms20092215

**Published:** 2019-05-06

**Authors:** Hamza El Hadi, Angelo Di Vincenzo, Roberto Vettor, Marco Rossato

**Affiliations:** 1 Internal Medicine 3, Department of Medicine DIMED, University of Padova, 35122 Padova, Italy; divincenzoang@gmail.com (A.D.V.); roberto.vettor@unipd.it (R.V.); marco.rossato@unipd.it (M.R.); 2 Department of Medicine, Klinikum Rheine, 48431 Rheine, Germany

**Keywords:** non-alcoholic fatty liver disease, cardio-metabolic disorders, hypertension, diabetes, dyslipidemia, chronic kidney disease, cardiac arrhythmia, ischemic stroke

## Abstract

With the progressive epidemics of obesity, non-alcoholic fatty liver disease (NAFLD) has become the most common cause of chronic liver disease in adults and children. The increasing prevalence and incidence of NAFLD with advanced fibrosis is concerning because patients appear to experience higher non-liver-related morbidity and mortality than the general population. Recent clinical evidence suggests that NAFLD is directly associated with an increased risk of cardio-metabolic disorders. This mini review describes briefly the current understanding of the pathogenesis of NAFLD, summarizing the link between NAFLD and cardio-metabolic complications, focusing mainly upon ischemic stroke, type 2 diabetes mellitus (DM), hypertension, chronic kidney disease (CKD) and cardiac arrhythmias. In addition, it describes briefly the current understanding of the pathogenesis of NAFLD.

## 1. Introduction

Non-alcoholic fatty liver disease (NAFLD) is the most common form of liver disease and a leading cause of morbidity and mortality in both developed and developing countries [1]. A large body of literature currently suggests that NAFLD is not only confined to the liver but might rather represent a major part of a multisystemic disease. As early as 1995 it was first suggested that NAFLD was a systemic condition with a specific cardio-metabolic involvement [2], a notion which is now universally accepted. As is well known, NAFLD patients usually die of extra-hepatic causes, frequently for cardiovascular diseases (CVD), which sustains the importance of an early diagnosis and a prompt treatment of CVD risk factors. There is abundant evidence of a direct link between NAFLD and multiple cardio-metabolic disorders including ischemic stroke, insulin resistance, hypertension, chronic kidney disease (CKD) and cardiac arrhythmias [3]. The current mini review will briefly highlight the current understanding of the pathogenesis of NAFLD, describing the association between NAFLD and cardio-metabolic disorders and discussing the underlying pathogenic mechanisms.

## 2. NAFLD: Definition, Epidemiology and Pathogenesis

In addition, NAFLD is a condition characterized by different hepatic abnormalities, ranging from simple liver steatosis to cirrhosis, with an increased risk for the development of hepatocellular carcinoma (HCC). The diffusion of the disease has reached epidemic levels in the last few decades, with an increased prevalence overlapping the spread of obesity and metabolic syndrome worldwide. As a consequence, NAFLD actually represents the most common chronic liver disorder, with a global prevalence of about 24%. Current data estimate that NAFLD affects 30% of the United States, 30% of the South American, 27% of the Asian, 24% of the European, 32% of the Middle East and 13% of the African population [1,4,5]. Today, this condition poses a relevant problem for all health systems because of the high prevalence of cardio-metabolic comorbidities and high liver-related mortality observed in these patients.

Non-alcoholic fatty liver (NAFL) or simple steatosis (a condition characterized by ≥5% hepatic steatosis without evidence of hepatocellular injury) is the starting point of NAFLD, and the majority of patients show this pattern [6]. Liver steatosis is usually considered as a benign condition, but a significant percentage of these patients’ conditions will evolve to liver fibrosis through via non-alcoholic steatohepatitis (NASH) [7], which is defined by the presence of histological abnormalities such as hepatocyte ballooning and lobular necro-inflammation, which may progress to irreversible damage [8]. Remarkably, the progression from NASH to fibrosis is associated with some predisposing factors, such as arterial hypertension, obesity and type 2 diabetes mellitus (DM) and, as a unique in liver pathology, HCC in these patients may develop in NASH also in the absence of liver cirrhosis. The risk of neoplastic degeneration in NAFLD patients is different according to the different population study [9]. Cumulative incidence ranges from 0.25% to 7.6% at 5 years in subjects with advanced fibrosis or established cirrhosis [10]. Several risk factors have been associated with an increased risk for neoplastic progression. Patatin-like phospholipase domain-containing protein-3 (PNPLA3) polymorphisms, elderly status, metabolic abnormalities and drugs may modulate the risk of developing HCC.

According to the current guidelines, the diagnosis of NAFLD may be performed with ultrasound, although it has limited sensitivity for people with a low degree of steatosis (<20%) and for individuals with a high body mass index (BMI) (>40 kg/m^2^). To this regard, liver biopsy is the gold standard for NAFLD diagnosis but it is impractical as a diagnostic tool as it is invasive and expensive. Currently, liver biopsy is implied in the histological definition and assessment of fibrosis in NASH patients [11]. Proton magnetic resonance spectroscopy represents the best method for an accurate quantification of liver fat accumulation, but it is applied only in the context of clinical trials and experimental purposes. In clinical practice, abnormal levels of hepatic transaminases are used for the diagnosis despite their elevation being non-specific and not correlating with the severity of fibrosis.

The pathophysiology of NAFLD is complex and multifactorial, involving different risk factors, both genetic (in particular, polymorphisms of the PNPLA3 gene) and environmental factors (Western diet, low physical activity), which probably act in a different manner along with the different phases of the disease, leading to both liver-specific and extra-hepatic manifestations. To this respect, a growing interest has generated the observations that NAFLD seems independently associated with an increased risk of CVDs, and cardiovascular complications are probably the main cause of death in these patients [3].

The strong epidemiological association with obesity and type 2 DM has led to considering NAFLD as an additional manifestation in the context of insulin resistance, despite the underlying mechanisms remaining not completely understood. The severity of obesity seems positively correlated with the degree of the histological pattern of NAFLD, as it can be observed by the high rates of NASH and advanced fibrosis up to cirrhosis in morbidly obese patients. In fact, while in non-obese subjects the prevalence of NAFLD is 16%, about two-thirds of patients with liver steatosis are overweight or obese [12], and in this population weight loss has been shown to be the most effective strategy for NAFLD improvement. Bariatric surgery seems the most effective approach for weight loss, although NAFLD is not an indication to surgery despite its high prevalence in severe obesity (BMI>40), ranging from 84% for NAFL to about 62% for NASH [13].

Considering the independent burden associated with the disease, recent recommendations suggest performing a screening for NAFLD in all subjects with metabolic syndrome and/or obesity to prevent possible complications, as the majority of patients with liver steatosis and even NASH are asymptomatic [14]. Moreover, a growing body of evidence emphasizes the importance of a broader patient assessment and demonstrates that NAFLD is a disease with systemic pathophysiologic consequences. Therefore, in patients with NAFLD a clinical evaluation with the assessment of blood pressure, waist circumference, BMI, plasma cholesterol, triglyceride levels, plasma glucose or glycosylated haemoglobin estimated that glomerular filtration rate (eGFR) and albuminuria is fundamental to recognize the presence of concomitant cardio-metabolic complications [15].

The hypothesis that was proposed to explain the interplay between NAFLD and metabolic disease has been modeled considering the effect of a hypercaloric diet (and also reduced physical activity) on fat mass. In obesity and metabolic syndrome, the excessive calorie intake promotes an expansion of adipose tissue with dysfunctional adipocytes and an increased triglycerides hydrolysis and a consequent increase of circulating free fatty acids (FFA). The initial phase in the development of NAFLD results from the increased uptake of FFA by the hepatocytes, with consequent liver steatosis [16].

The role of visceral adiposity as a better predictor of NAFLD rather than body weight and BMI [16,17] is progressively increasing. Several studies have suggested a high correlation between visceral adiposity and the development of fatty liver [18,19]. An increase in visceral fat results in increased triglycerides hydrolysis and consequent FFA delivery to the liver, which in turn is closely related to the development of insulin resistance.

In recent years, it has become increasingly obsolescent to regard the so called NAFLD “two hit hypothesis”, considering the fact that insulin resistance as the “first hit” induces lipid accumulation within the hepatocytes, thus increasing the vulnerability of the liver to further insults—referred to as the “second hit” that in turn promoted hepatic injury, inflammation and fibrosis. Today, a “multiple parallel hits hypothesis” seems to provide a more accurate explanation of NAFLD pathogenesis [20].

**Figure 1:**
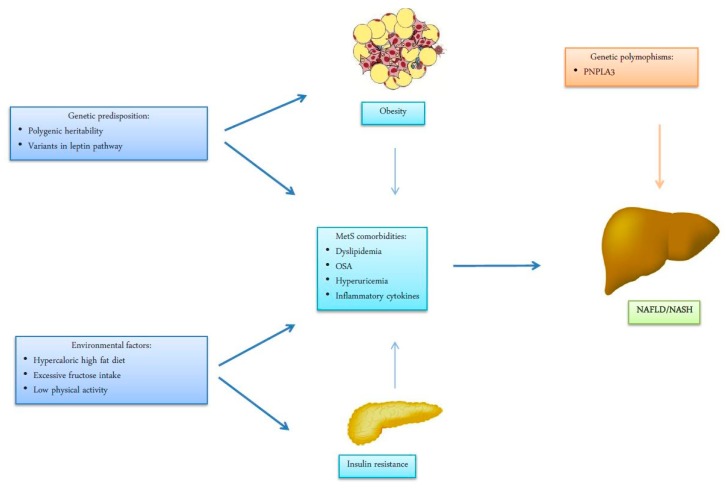
Environmental factors, life style and genetic susceptibility have shown to be implicated in the pathogenesis of visceral obesity and insulin resistance. The metabolic and respiratory complications associated with obesity and insulin resistance as dyslipidemia, OSA (obstructive sleep apnea) Hyperuricemia, hormonal imbalance and systemic low-grade inflammation are established factors in the pathogenesis of non-alcoholic fatty liver disease (NAFLD) and progression towards non-alcoholic steatohepatitis (NASH). Remarkably, genetic variation in the patatin-like phospholipase domain-containing protein-3 (PNPLA3) gene is considered a prominent risk factor for progression of liver injury from steatosis to steatohepatitis, cirrhosis and hepatocellular carcinoma (HCC).

In the model of a “multiple hit process”, adipocyte dysfunction in genetically predisposed subjects is associated with a concomitant reduction of insulin sensitivity which is responsible for a reduced liver fatty acid oxidation and intra-hepatic accumulation of triglycerides (IHTG). The contribution to insulin resistance is due to the abnormal production of adipose-derived cytokines and hormones, such as tumor necrosis factor alpha (TNF-α), interleukin (IL)-6, C-reactive protein (CRP), leptin, adiponectin and resist in, with the induction of an inflammatory pathway which amplifies liver lipotoxic damage. In fact, from adipocyte-derived inflammation the condition evolves to an intrahepatic inflammation with hepatocyte injury, which is responsible for the progression of liver disease to NASH and cirrhosis (Figure 1). Increased levels of FFA, IHTG and the increased production of diacylglicerol and other lipotoxins activate protein kinase C-delta (PKC-δ) and nuclear factor kinase-B (NF-κB), leading to liver inflammation and insulin resistance. These hits also contribute multiple nutritional factors and gut microbiota [20,21].

In addition, other obesity-associated comorbidities, such as obstructive sleep apnea [22], hyperuricemia and even hypo-testosteronemia in men and polycystic ovary syndrome in women [23] have been associated with the development of NAFLD, suggesting a more complex interaction between obesity and liver function (Figure 1).

## 3. Association between NAFLD and Cardio-Metabolic Disorders

### 3.1. Ischemic Stroke in NAFLD

Ischemic stroke is one of the leading causes of mortality and long-term disability. The main initiating event consists in an impairment of blood flow in a portion of the brain, mainly due to occlusion of an intracranial or neck blood vessel. Emerging evidence suggests a relationship between ischemic stroke and biomarkers of NAFLD. In a cross-sectional study including 103 patients with acute ischemic stroke diagnosed by magnetic resonance tomography (MRT) and 200 healthy controls, elevations of alanine aminotransferase (ALT) or aspartate aminotransferase (AST) have been shown to be independently associated with ischemic stroke [24]. In a case-control study performed considering ongoing cohort studies in three European countries (United Kingdom, Netherlands and Finland), the incidence of ischemic stroke has been shown to be strongly associated with increased gamma-glutamyl transferase (GGT) levels [25]. GGT is known to be one of the main contributors in atherosclerosis and subsequent cerebrovascular diseases, mainly by inducing production of pro-inflammatory mediators and increasing the release of reactive oxygen species (ROS) [26]. In a recent study, NAFLD has been shown to be associated with an increased prevalence of lacunar infarcts in non-obese subjects [27]. A recent meta-analysis evaluating nine case-control studies showed that NAFLD is related to a higher risk of ischemic stroke even after adjustment for cardiovascular risk factors such as obesity, dyslipidemia and type 2 DM [28]. In a two-year prospective study including 200 patients with acute ischemic stroke, NAFLD (based on a combination of ultrasound findings and elevated aminotransferase levels) was found in 42.5% of the study population. In addition, patients with NAFLD have been shown to be associated with a worse clinical outcome at the moment of hospital discharge. However, these verity of stroke in the study population was not adjusted for the main cardiovascular risk factors such as obesity, dyslipidemia and type 2 DM [29]. Similarly, it has been shown that NAFLD is associated with an increased prevalence of brainstem infarctions. In this retrospective study, NAFLD was an independent determinant of the severity and progression of acute brainstem infarctions after adjustment of other risk factors, such as age, gender, DM and inflammatory markers [30].

### 3.2. Arterial Hypertension in NAFLD

Arterial hypertension is considered a major cardiovascular risk factor and represents a leading contributor to stroke and ischemic heart disease. In a large prospective cohort of Korean men, baseline NAFLD, assessed by ultrasonography, was independently associated with an increased incidence rate of hypertension. In addition, this rate was potentially associated with different degrees of NAFLD [31].

In another five-year prospective study, the incidence of NAFLD was associated with increased odd ratios for incident hypertension even after adjustment of potential risk factors for hypertension. In this study, individuals improving fatty liver during the follow-up period presented the same risk of incident hypertension compared with subjects without NAFLD, whereas the resolution of fatty liver during the same period was not associated with a significant increase in incident hypertension, suggesting that the change in fatty liver status overtime could influence the incidence of hypertension [32].

In another observational study including participants randomly selected from the general population, NAFLD was independently associated with prevalent hypertension and high-normal systolic blood pressure [33].

Moreover, a large study comparing the prevalence of NAFLD in lean and overweight-obese individuals using ultrasonography and its association with other cardio-metabolic disorders reported a higher incidence of hypertension in lean individuals with NAFLD. These results suggested that NAFLD seems to be related to a higher cardiovascular risk in normal weight patients [34].

In actuality, it is believed that the relationship between NAFLD and hypertension is bidirectional. This was first reported in two large cohort prospective studies that investigated the causality between incident NAFLD and metabolic syndrome components. In these studies, arterial hypertension was found to be an independent predictor for ultrasound-diagnosed NAFLD [35,36].

In another large cohort study on Brazilian individuals, hypertension was independently associated with an increase in the odds of prevalent NAFLD diagnosed by ultrasound. In addition, using the noninvasive fibrosis risk score (FIB-4), it was reported that the optimal control of blood pressure protects against moderate-to-severe hepatic fibrosis risk [37].In another cross-sectional study among hypertensive patients with NAFLD proven by biopsy, the use of renin-angiotensin-aldosterone system (RAAS)inhibitors combined with either an angiotensin converting enzyme inhibitor (ACE-I) or an angiotensin receptor blocker (ARB) was associated with less advanced hepatic fibrosis compared with the control group [38].

Recently, this bi-directional relationship was observed also in the prospective cohort study of the Framingham Heart Study over a six-year of follow-up in a group of middle-aged to older adults.

NAFLD at baseline, assessed by multiple-detector computed tomography, was associated with increased odds of incident hypertension. Paralleling this, arterial hypertension was associated with an increased risk of developing NAFLD [39].

### 3.3. Diabetes in NAFLD

Diabetes is one of the most common metabolic disorders in the world. The link between NAFLD and type 2 DM is complex, since both disorders share multiple common pro-inflammatory and pro-fibrotic pathways [40,41]. Several extensive studies have indicated that more than 70% of patients with type 2 DM develop NAFLD [42,43].

Two large meta-analyses reported that NAFLD is associated with an increased risk of incident DM. In both meta-analyses, most included observational studies relying on abnormal liver enzymes to identify patients with NAFLD [44,45].Similar findings have been reported using non-invasive imaging techniques to diagnose NAFLD, such as ultrasonography or computed tomography (CT) [46,47].

Besides type 2 DM, NAFLD has been shown to also be associated with impaired fasting glucose [48]. In a large cross-sectional study, NAFLD diagnosed by ultrasound scan was shown to be an independent risk factor for incident diabetes under conditions of impaired insulin secretion [49]. In a five-year prospective study, the degree of hepatic steatosis was shown to be positively correlated with the future development of type 2 DM, even after adjustment for risk factors [50].

Increasing evidence from studies with biopsy-diagnosed NAFLD suggest that the coexistence of type 2 DM represents an independent predictor of histological severity and progression in advanced fibrosis of NAFLD [51,52,53]. Further investigations based on liver histology reported that patients with type 2 DM have more severe NAFLD than those without type 2 DM, with a higher prevalence of NASH (80.2% vs 64.4%, respectively) and advanced fibrosis (40.3% vs 17.0%, respectively) [54]. Despite the debate concerning the causality between DM and NAFLD, increasing epidemiological evidence suggests that there is a bidirectional relationship between NAFLD and type 2 diabetes and that NAFLD may precede the development of type 2 DM [40,41,55]. Therefore type 2 DM screening should be suggested and implemented in patients with NAFLD and/or advanced liver fibrosis.

Since insulin resistance is considered as a main pathogenetic factor for the development of NAFLD, pharmacological strategies to improve insulin sensitivity have been investigated as potential treatment options for NAFLD, but the studies have yielded conflicting results [56].

Metformin, the most widely used insulin-sensitizing agent, improves the biochemical and metabolic features of NAFLD. However, it showed a scarce positive impact on histological markers associated with NAFLD [57].

Pioglitazone is another family of insulin-sensitizing agents that have been associated with improvements in histological findings in NAFLD [58]. However, concerns about the side effects and long-term safety of these molecules have limited their widespread use in NAFLD and NASH treatment.

Glucagon-like peptide-1 (GLP-1) agonists represent a novel class of antidiabetic drugs. They have been shown to be effective in improving liver histology and reducing aminotransferase levels in patients with NASH [59]. However, the evidence of an actual effectiveness of GLP-1 receptor agonist in the treatment of NAFLD is still lacking.

The most recent drugs introduced in the treatment of diabetes are the sodium glucose cotransporter type-2 (SGLT2) inhibitors, a class of glucose-lowering agents improving glucose control by excreting glucose with the urine. These agents also exert important cardiovascular protection in type 2DM patients with established cardiovascular disease. Recent studies have shown that the treatment of diabetic patients with SGLT2 inhibitors reduces fatty liver content, as assessed by different imaging techniques, also improving biological markers of NAFLD [60,61].

### 3.4. Dyslipidemia in NAFLD

As previously mentioned, abnormal lipid metabolism is a cause and a consequence of NAFLD, and the development of dyslipidemia may further account for the CV risk observed in these patients [62].

Excessive intra-hepatic fat deposition characterizing hepatic steatosis is promoted by the increased circulating levels of FFA (resulting from an enhanced lipolysis within adipose tissue), finally resulting in very low-density lipoprotein (VLDL) overproduction. Hepatic FFA uptake is the key point for increased triglycerides synthesis, resulting from the balance between circulating FFA and hepatocytes uptake capacity deriving from the activity of membrane transporters (fatty acid transport proteins). Patients with NAFLD show an increased FFA uptake, leading to an abnormal “de novo lipogenesis” and higher triglyceride levels. Reduced mitochondrial oxidation of FFA is the second step of this process, responsible for the overall reduced clearance of several lipoproteins.

Lipid accumulation within hepatocytes is responsible for the initial hepatic damage, and then lipotoxicity, oxidative stress, mitochondrial dysfunction and the induction of inflammatory cascade with the expression of TNF-α, IL-1 and IL-6 promote the progression to the advanced stages of the disease. To this respect, it is interesting to note that the severity of serum lipid profile seems related to the degree of hepatic injury, with higher disturbances in NASH [63].

Lipid profile in NAFLD is characterized by decreased levels of high-density lipoprotein (HDL)- and increased levels of low-density lipoprotein (LDL)-cholesterol and triglycerides, and even by a high apolipoprotein B to apolipoprotein A-1 ratio. In particular, NAFLD has been independently associated with small dense LDL particles, resulting in a more atherogenic profile [64]. This condition reflects the qualitative derangements of lipid profile observed in NAFLD—hypertriglyceridemia activates the cholesteryl-ester-transfer protein, enhancing the synthesis of these triacylglycerol-rich LDL particles.

NAFLD also seems to be associated with lower levels of HDL, which are usually considered to have a high anti-atherogenic potential [65].

### 3.5. Chronic Kidney Disease in NAFLD

CKD is increasingly regarded as a health problem worldwide and is considered an independent cardiovascular risk factor leading to decreased quality of life and premature mortality [66]. It is defined by the presence of renal dysfunction, as measured by the estimated eGFR (derived from serum creatinine using standard estimating equations) and abnormal albuminuria or overt proteinuria [66]. Evidence accumulated in the last few years suggests a direct relationship between NAFLD and CKD. It has been estimated that the prevalence of CKD in NAFLD patients, diagnosed either by imaging or histology, ranges approximately from 20% to 55%, compared with5% to30% in patients without NAFLD [67].

In a recent meta-analysis including twenty studies (11 cross-sectional and 9 longitudinal), NAFLD patients were associated with an increased risk and severity of prevalent and incident CKD, even after adjustment for significant risk factors. Interestingly, NASH and advanced fibrosis were also associated with higher odd ratios for proteinuria and with a lower eGFR than simple hepatic steatosis and non-advanced fibrosis, respectively [68].

These findings were further confirmed by several case-control studies that utilized liver biopsy to diagnose NAFLD/NASH [69,70,71,72,73]. These studies have shown that patients with histologically defined NAFLD/NASH have lower eGFRs and a higher frequency of CKD or abnormal albuminuria than matched controls. The severity of NAFLD or NASH was positively correlated with that of CKD regardless of other components of the metabolic syndrome.

### 3.6. Cardiac Arrhythmias in NAFLD

Recent clinical investigations revealed a significant increase of cardiac arrhythmias among patients with NAFLD, suggesting that NAFLD could represent a risk factor for various types of arrhythmias including atrial fibrillation, cardiac conduction defects and ventricular arrhythmias.

Two large population-based cohort studies described the association between mildly elevated liver enzymes (ALT and AST), as markers of NAFLD, and atrial fibrillation (AF). Furthermore, it has been reported that elevated AST levels were independently associated with increased long-term risk of incident AF [74,75].

The diagnosis of NAFLD by ultrasonography has also been associated with an increased prevalence and incidence of AF in a hospital-based sample of patients with type 2 DM, independently from several clinical risk factors for atrial fibrillation such asage, sex, arterial hypertension and electrocardiographic features [76,77].These findings were later confirmed by another prospective study showing that NAFLD was independently associated with an increased risk of incident AF over a mean follow-up of 16 years [78].

In a recent meta-analysis of five observational cohort studies (two cross-sectional and three longitudinal studies) with 238,129 participants, NAFLD assessed by ultrasonography was associated with a nearly two-fold increase in the prevalence and incidence of atrial fibrillation [79].

Abnormal prolongation of the electrocardiographic corrected QT interval (QTc) predisposes to malignant ventricular arrhythmias and sudden cardiac death. In a recent cross-sectional study considering nearly 31,000 patients, the presence and severity of NAFLD was associated with an increased risk of QTc interval prolongation in patients with and without type 2 DM [80].

A retrospective study reported that patients with NAFLD, when compared with patients without NAFLD, had a nearly three-fold increased risk of ventricular arrhythmia demonstrated by 24-hour Holter monitoring (defined as more than 30 premature ventricular contractions per hour, non-sustained ventricular tachycardia or both). This association also remained consistent after adjusting for different cardiovascular risk factors, comorbidities and the use of various medications [81].

In the same context, patients with ultrasound-diagnosed NAFLD showed a three-fold increase risk of persistent heart block (defined as at least one block among first-degree a trio-ventricular block, second-degree block, third-degree block, left bundle branch block, right bundle branch block, left anterior hemi-block or left posterior hemi-block) compared with those without NAFLD. Notably, this risk was positively correlated with the severity of liver disease even after adjustment for age, gender, hypertension and prior ischemic heart disease, among other potentially confounding factors [82].

## 4. Mechanisms Linking Cardio-Metabolic Disorders with NAFLD

The mechanisms underlying the possible independent association between NAFLD and cardio-metabolic disorders have not been fully elucidated yet. In the last decade the link between these two conditions has been a hot topic in basic and clinical research.

Low-grade inflammation characterizes metabolic disorders, such as NAFLD and visceral obesity which is mainly determined by the expansion of ectopic abdominal fat, which leads to an increased release of pro-inflammatory cytokines that could crucially contribute to the development of insulin resistance and other NAFLD-related extrahepatic complications [83]. Insulin resistance, a characteristic feature in NAFLD, has been suggested to promote the progression of CKD by worsening renal haemodynamics, increasing sodium retention and the activation of the sympathetic nervous system [84].

As mentioned in the previous section, the inflammatory process within the expanding white adipose tissue has been suggested to activate the pro-inflammatory NF-κB pathway in the liver and increase the transcription of a variety of pro-inflammatory genes that can amplify systemic chronic inflammation, thus leading to extrahepatic cellular injury [85].

Another feature in NAFLD is atherogenic dyslipidemia, which comprises a triad of elevated levels of triglycerides and small-dense low-density lipoprotein and low levels of high-density lipoprotein cholesterol. This represents a potential risk factor of endothelial dysfunction and reno-vascular damage [86]. NAFLD, especially in its necro-inflammatory form, is often associated with an increased release of pro-coagulant, pro-oxidant and pro-fibrogenic factors mediating additional organ dysfunction [87].It has been suggested that pro-inflammatory cytokines and different pro-thrombotic factors are associated with increased arrhythmogenic complications, possibly by cardiac structural and electrical remodeling [88].

NAFLD is associated with an increase in components of the renin–angiotensin system, such as angiotensin II, that can contribute to vascular damage via increases in oxidative stress and subsequently blocking the insulin-signaling pathways and accelerating atherosclerosis [89]. In addition, the impairment of insulin-signaling in the endothelium leads to vasoconstriction, thus promoting arterial hypertension [90]. Angiotensin II also accelerates the progression of NAFLD to NASH and then to fibrosis by stimulating fibroblasts and inducing the release of pro-inflammatory cytokines [91].

Recent data support a role for adiponectin in mediating the cardio-metabolic complications in patients with NAFLD. Adiponectin is an adipocyte-derived anti-inflammatory and anti-atherogenic mediator. Patients with NAFLD have shown reduced circulating adiponectin concentrations independent of other metabolic confounding factors [92]. The regulation of adiponectin levels have been shown to be mediated by fetuin-A, a liver-secreted protein that is strongly correlated with fatty liver, impaired glucose tolerance and insulin resistance [93]. Decreased plasma adiponectin levels lead to a suppression of 5’ adenosine monophosphate-activated protein kinase (AMPK)-activation, thereby stimulating pro-inflammatory and pro-fibrogenic cascades in hepatocytes and podocytes, possibly causing liver and kidney damage [94].

There is growing evidence that the imbalance of the intestinal microbiota, termed dysbiosis, is involved in the pathogenesis and severity of hepatic steatosis [95]. This is usually associated with increased intestinal permeability by disrupting intracellular tight junctions leading to the release of lipopolysaccharide (LPS), cytokines and gut microbiota DNA into the circulation and to the liver, promoting hepatic and systemic inflammation. Dysbiosis also promotes the increased production of secondary bile acids that, in turn, have been linked to toxic effects on mitochondrial and cell membranes [96].

Different molecules generated by intestinal microbiota (such as trimethylamine, p-cresol and indole) have been described after further metabolism within the liver to promote atherosclerosis, probably through the upregulation of multiple macrophage scavenger receptors [79].

Additionally, an increase of systemic levels of trimethylamine-N-oxide (TMAO) has shown to be associated with poor clinical outcomes, probably by promoting tubule-interstitial fibrosis in the kidney [97].

## 5. Conclusions

There is now strong evidence that NAFLD is a systemic disease that contributes to the development and progression of extra-hepatic diseases such as type 2 DM, CKD, atherosclerosis, arterial hypertension, cardiovascular diseases and cardiac arrhythmias. Therefore, these data imply that patients with diagnosed NAFLD should be screened at an earlier stage for associated cardio-metabolic disorders and a multi-disciplinary-team-based approach should be involved in the management and treatment of these patients.

Currently, the histopathological examination of liver biopsy represents the gold standard for diagnosis of NAFLD. As a result, the non-invasive assessment of hepatic steatosis using serological tests and radiological imaging can be associated with substantial false positive and false negative rates. Therefore, the lack of standardized diagnostic methods in NAFLD makes the determination of the relationships between NAFLD and cardiometabolic disorders even more challenging.

The causal factors linking NAFLD to cardiometabolic complications remain to be definitively established. Therefore, more research is required to elucidate the pathophysiological links between NAFLD and these systemic complications. This will possibly lead to the development of new therapeutic approaches for NAFLD, thus leading also to a decrease of the global burden of related cardio-metabolic diseases.

Furthermore, more prospective and intervention studies of well-characterized patients with NAFLD are needed to definitively prove whether the improvement or resolution of NAFLD can prevent the development of cardio-metabolic risks.

## Abbreviations

ACE-I	angiotensin converting enzyme inhibitor
AF	atrial fibrillation
ALT	alanine aminotransferase
AMP	adenosine monophosphate
AMPK 5′	AMP-activated protein kinase
ARB	angiotensin receptor blocker
AST	aspartate aminotransferase
BMI	body mass index
CKD	chronic kidney disease
CRP	C-reactive protein
CT	computed tomography
CVD	cardiovascular diseases
DM	diabetes mellitus
eGFR	estimated glomerular filtration rate
FFA	free fatty acids
GGT	gamma-glutamyl transferase
HCC	hepatocellular carcinoma
HDL	high-density lipoprotein
IHTG	intra-hepatic accumulation of triglycerides
IL	interleukin
LPS	Lipopolysaccharide
LDL	low-density lipoprotein
MRT	magnetic resonance tomography
NAFL	non-alcoholic fatty liver
NAFLD	non-alcoholic fatty liver disease
NASH	non-alcoholic steatohepatitis
NF-κB	protein kinase C-delta (PKC-δ) and nuclear factor kinase-B
OSA	obstructive sleep apnea
PNPLA3	patatin-like phospholipase domain-containing protein-3
QTc	corrected QT interval
RAAS	renin-angiotensin-aldosterone system
ROS	reactive oxygen species
SGLT2	sodium glucose cotrasporter type-2
TMAO	trimethylamine-N-oxide
TNF-α	tumor necrosis factor alpha
VLDL	very low-density lipoprotein
